# Personality, Risk Perceptions, and Health Behaviors: A Two-Wave Study on Reciprocal Relations in Adults

**DOI:** 10.3390/ijerph192316168

**Published:** 2022-12-02

**Authors:** Cecilie Thøgersen-Ntoumani, Andreas Stenling, Esther Izett, Eleanor Quested

**Affiliations:** 1 Danish Center for Motivation and Behavior Science, Department of Sports Science and Clinical Biomechanics, University of Southern Denmark, 5230 Odense, Denmark; 2 Department of Psychology, Umeå University, 90187 Umeå, Sweden; 3 Department of Sport Science and Physical Education, University of Agder, 4630 Kristiansand, Norway; 4 Curtin School of Population Health, EnAble Institute, Curtin University, Perth, WA 6102, Australia

**Keywords:** physical activity, eating behaviors, illness risk perceptions, personality

## Abstract

The aim of the study was to examine reciprocal associations between risk perceptions for cardiovascular disease and health behaviors (i.e., physical activity, fruit/vegetable consumption), while accounting for key personality characteristics in middle-aged adults. Participants (*N* = 297; *M* (*SD*) age = 51 (6.95); 72.4% female) completed online questionnaires assessing risk perceptions, physical activity, fruit/vegetable consumption, and personality (conscientiousness and neuroticism) on two occasions, one week apart. Physical activity did not have a statistically significant effect on risk perception over time (*b* = −0.00, *p* = 0.227). However, fruit and vegetable consumption (*b* = −0.19, *p* = 0.006) and neuroticism (*b* = 0.22, *p* = 0.001) predicted risk perception. Risk perception did not have a significant effect on physical activity (*b* = −343.86, *p* = 0.147) or fruit/vegetable consumption (*b* = −0.08, *p* = 0.144) over time; however, neuroticism had significant and negative effects on physical activity (*b* = −520.84, *p* = 0.029) and fruit/vegetable consumption (*b* = −0.20, *p* = 0.001). High levels of neuroticism in middle age may hinder engagement in physical activity and consumption of fruit/vegetable behaviors and should therefore be targeted accordingly to increase protective health behaviors and reduce incidence of cardiovascular disease.

## 1. Introduction

Cardiovascular disease (CVD) accounts for 31% of deaths worldwide [1], and 27% of deaths in Australia [2]. Rates of CVD incidence have a large economic impact and reduce quality of life and psychological wellbeing [3]. Meeting recommended levels of physical activity (PA) and consuming adequate amounts of fruit and vegetables reduces the risk of CVD [4,5]. However, survey findings from developed countries, including the United States [6], the United Kingdom [7], and Australia [8] have shown that PA levels and rates of fruit and vegetable consumption amongst adults are well below the recommended guidelines. The highest rates of CVD incidence occur amongst those aged 40 to 65 years who also report low rates of PA and fruit and vegetable consumption [2].

Health psychology research has focused on investigating psychological factors that influence health behavior change to reduce incidence of chronic disease such as CVD [9]. Risk perception, also referred to as perceived susceptibility, is an individual’s subjective appraisal of the likelihood of developing a disease, and is a central concept in many health behavior change theories (e.g., it is one of the key constructs in the health belief model (HBM) [10]. When individuals perceive their risk of disease to be high, they are more likely to take preventative action to improve their health, particularly if they believe that they have control over their risk of developing the disease. Baseline data from a large trial (*N* = 2362) with healthy adults found that CVD was considered the most controllable disease compared to other diseases [11].

The behavior motivation hypothesis proposes that perceptions of high risk of a disease will motivate engagement in relevant health behaviors [12]. Accordingly, this would translate to risk perceptions positively predicting health behaviors including physical activity and healthy eating. However, support for the utility of risk perception in the prediction of health behavior has been mixed. A meta-analysis (*k* = 18), which examined longitudinal associations between constructs from the HBM and health behavior, found a near zero effect [13]. Only one of the studies included in the analysis examined exercise, and none examined diet-related behavior. Carpenter’s results contrast with a more recent meta-analysis (*N* = 208; *k* = 239) of experimental studies [14]. Sheeran et al. found that most interventions (12/15) which successfully changed risk perceptions resulted in increased intentions (*d*_+_ = 0.31) to engage in over 15 health and risk behaviors (e.g., smoking, vaccination, alcohol consumption, exercise, and diet), and about half of the studies (8/14) showed that heightened risk perceptions fostered health behavior change (*d*_+_ = 0.23). However, only six independent effects of risk perceptions on exercise behavior were available, and five for diet behaviors.

Engagement in health behaviors may also influence risk perceptions, as postulated by the “accuracy hypothesis” [15]. According to this hypothesis, individuals can more accurately judge perceived risk of disease if they engage in relevant health behaviors. We are aware of only one longitudinal study that has examined whether bi-directional, or reciprocal, associations exist between risk perceptions and health behaviors. A 5-year prospective study was conducted with Finnish adults (*N* = 909) with perceived low or high risk of type 2 diabetes or perceived low or high CVD risk [16]. A cross-lagged autoregressive model showed that risk perception (relating to both type 2 diabetes and CVD) did not predict PA 5 years later (time 2), nor did PA at time 1 predict perceptions of risk at time 2 [16]. Five years may be too long to observe effects between the variables. There could be value in examining these associations using shorter timeframes as it is less likely that the associations are confounded by extraneous variables. Further, results of Carpenter’s meta-analysis found that the longer the time between measurements of risk at time 1 and behavior at time 2, the weaker the association between the two constructs [14]. Further, indices of diet were not examined in the study by Vornanen et al. [16].

To extend the work of Vornanen and colleagues, it may also be pertinent to consider personality traits, given their associations with both risk perception [17] and health behaviors [18]. In terms of risk perception, it has been argued that personality may affect how health adherence interventions are received [19]. Specifically, neuroticism and conscientiousness are the most consistent personality predictors of both PA and dietary behaviors [18,20,21,22]. Individuals high in conscientiousness are described as competent and proactive whilst individuals high in neuroticism are considered irritable and unstable [18]. Individuals with higher levels of conscientiousness perceive themselves to be at lower risk of disease (e.g., CVD, breast cancer, osteoporosis, and diabetes [17,19]). In contrast, people with high levels of neuroticism tend to perceive themselves at greater risk of disease, including CVD [19].

Regarding health behaviors, conscientiousness positively predicts PA engagement, whilst neuroticism is positively associated with sedentary behaviors [18]. Furthermore, conscientiousness positively predicts fruit and vegetable consumption while a poorer quality diet has been associated with higher levels of neuroticism [23]. The direct influences of personality on behavior and risk perception are thought to be due to the traits inherent in each personality type [18]. Conscientiousness involves discipline, self-awareness, and caution, which promote positive health behaviors and realistic appraisals of health threats [24]. In contrast, higher levels of worry and instability reported in people high in neuroticism are thought to explain reluctance to engage in health behaviors, impulsivity in poorer diet choices and general anxiety towards one’s health [18].

Limitations are associated with existing research on risk perceptions. Single item scales have generally been used to measure risk perceptions, failing to capture the proposed multi-dimensionality of the construct [25]. Ferrer and Klein suggested that perceived risk should be categorized into affective, experiential, and deliberative dimensions, proposing the Tripartite Model of Risk Perception. Affective risk perceptions refer to the emotional, affective responses to risk, experiential risk perceptions are gut-level and heuristic-based appraisals, and deliberative risk perceptions are thought to be the logic-based perception of the likelihood of developing disease [26]. A more comprehensive approach to measuring risk perception may account for more variance in the construct and improve the predictive utility of risk perception within health models [25]. Ferrer et al. [26] developed a measure to capture the three dimensions of risk perceptions; however, research on its psychometric properties is scarce beyond this initial study. Thus, further research is warranted to provide additional psychometric evidence and extend generalizability of this instrument. Finally, risk perception research has focused extensively on relatively simple, short-term behaviors such as screenings, vaccines, or condom usage as opposed to long-term changes in more complex behaviors (e.g., PA and diet [27]). Due to the high rates of chronic non-communicable diseases, further investigation of lifestyle risk factors such as PA, diet, and their relationship with health beliefs should be conducted [3].

The current study will be the first to test reciprocal relations incorporating risk perceptions for CVD, PA, fruit/vegetable consumption, *and* key personality characteristics (conscientiousness and neuroticism) in middle-aged adults. The results of the study can provide much needed insight into the directionality of the associations between the variables. In turn, these findings could have implications for interventions designed to either foster increased engagement in PA and/or fruit/vegetable consumption or empower people who are physically active and who consume adequate amounts of fruit/vegetables by providing them with a more accurate perception of their CVD risk. Incorporating conscientiousness and neuroticism in our models will provide for a more nuanced understanding of psychological factors impacting risk perceptions and PA plus fruit/vegetable consumption.

We will control for age, gender, marital status, and prior condition (including prior heart surgery) because these constructs have all shown previous associations with CVD outcomes [9]. It is hypothesized that:

1. Risk perceptions and health behaviors will be reciprocally associated, such that perceptions of risk at time 1 (T1) will positively predict health behaviors (PA and fruit/vegetable consumption) at time 2 (T2). Further, health behaviors at T1 will negatively predict risk perceptions at T2.
2. Conscientiousness at T1 will positively predict PA and fruit/vegetable consumption, and negatively predict risk perception at T2.
3. Neuroticism at T1 will negatively predict PA and fruit/vegetable consumption and positively predict risk perception at T2.

## 2. Materials and Methods

### 2.1. Research Design

The study adopted a quantitative, prospective research design over a 1-week period. At T1, data were collected for all variables. Data for most variables, except demographics and personality (conscientiousness and neuroticism), were collected at T2, one week later. The research design allowed us to examine short-term prospective relations and the temporal separation of independent and dependent variables reduces the risk of common method bias [28].

### 2.2. Participants

Participants between 40 to 65 years of age were eligible to take part. In total, 435 (49% female) participants completed the survey at T1 and 267 (61%) participants completed the follow up survey at T2. The mean age of the final sample included in the analyses (*n* = 297) was 51 (*SD* = 6.95) years, of which 72.4% were female. The majority of the participants were in a relationship (77.8%) and 4.7% reported having a prior heart condition or heart surgery.

### 2.3. Procedure

Ethical approval was sought and received (HRE2019-0349) from the Human Research Ethics Committee at Curtin University, prior to commencement of data collection. Convenience sampling was used. Recruitment strategies included sharing the survey link via Facebook posts, distribution to church and school staff email lists, and word of mouth. Incentive raffle draws to win one of four $50 supermarket vouchers were used to encourage participation. A Facebook booster post was also used to target users. Participants completed the online open survey using Qualtrics, a web-based survey tool. Participants indicated their consent by clicking next to begin the survey from the consent instruction screen. After completing T1 of the survey, participants were asked to provide their email address to receive the link for the T2 survey 7 days later.

### 2.4. Measures

**Risk perceptions.** Affective, experiential, and deliberative risk were measured using the Tripartite Model of Risk Perception (TRIRISK [26]) adapted for heart disease. The 18-item scale has three subscales for the three types of risk, each containing six items. Example items for each include, “How worried are you about developing heart disease in the future?” (affective), “How concerned are you about developing heart disease in your lifetime?” (experiential), and “How likely is it that you will get heart disease at some point in the future?” (deliberative). Response formats vary according to each item; these include responses on a 7-point Likert-type scale (1–7) and an open-ended percentage numeric response. Responses on Likert-type scales include, “likely to unlikely”, “very low to very high”, “strongly disagree to strongly agree”, and “much lower to much higher”. Higher scores of each subscale indicate higher levels of perceived risk of CVD. Reliability estimates (McDonald’s ω) amongst the sample for the three subscales ranged from 0.86 to 0.97.

**Physical activity.** PA level was measured using the International Physical Activity Questionnaire Short Form (IPAQ-SF [29]). The seven-item questionnaire assessed levels of participation in different levels of PA intensity, which were used to calculate an overall PA score. The items asked participants to respond with a relevant numerical value (0–7) of how many days in the past 7 they had completed each activity. Activities include vigorous, moderate, walking, and sitting with two items per activity. The first item of each activity asked, “During the last 7 days, on how many days did you [walk] for at least 10 minutes at a time?”. Participants responding with more than 0 days were then asked to record how much time was spent on each activity in hour and/or minute form with higher scores indicating higher levels of PA. Hours and minutes data for each participant were converted into a total minute score and converted into metabolic equivalent of the task (MET) and used as a continuous variable. The IPAQ-SF has demonstrated good test-retest Spearman’s reliability (*r =* 0.32–0.65) as well as reasonable construct validity with objective PA measures (*r* = 0.51–0.64 [30]).

**Fruit and vegetable consumption.** Levels of fruit and vegetable consumption were recorded using two items from the Dietary Instrument for Nutrition Education (DINE; [31]). Participants were asked to respond to the question, “Thinking about the last 7 days, about how many times a day did you eat the following?” in relation to their fruit consumption and their vegetable consumption. A Likert-type scale ranging from 1 (no times) to 4 (6 times or more) was used. Higher scores indicate a higher level of consumption of fruit and vegetables and were analyzed as an average score. The DINE has shown good convergent validity with other objective measures of fiber intake (*r* = 0.46–0.52 [31]).

**Personality.** Conscientiousness and neuroticism were measured using the Australian Personality Inventory (API [32]). The 20-item questionnaire has 10 items for each construct. Participants were asked to respond regarding how accurately they believed a statement described themselves on a 5-point Likert-type scale ranging from 1 (very inaccurate) to 5 (very accurate) with a higher score indicating higher levels of agreement. Example items for neuroticism and conscientiousness are, “Dislike myself” and “Am always prepared” respectively. Reliability estimates (McDonald’s ω) for neuroticism and conscientiousness were 0.89 and 0.83, respectively.

**Demographics.** Age, gender, and relationship status were measured via single items. Participants were also asked if they took medication for high blood pressure or had a prior diagnosis of a cardiovascular condition, or if they had undergone cardiovascular surgery. Reponses were recorded using a yes or no format.

**Statistical Analysis.** First, we examined the factor structure of the TRIRISK model scale. Following Ferrer et al. [26], we specified a three-factor independent clusters model confirmatory factor analysis (ICM-CFA), but also explored other factor structures due to the poor fit of the three-factor ICM-CFA model. The specification of zero cross-loadings on non-target latent factors in the ICM-CFA often results in poor model fit and inflated factor correlations [33,34]. Second, we compared the CFA model with a three-factor exploratory structural equation model (ESEM) in line with current recommendations [35]. Most items are imperfect to some degree and have some systematic association with other constructs; hence, cross-loadings as implemented in ESEM can typically be justified based on substantive theory or item content in multidimensional measures [33]. ESEM also shows more accurate latent factor correlations than ICM-CFA, which have been shown in both simulated and empirical data [33,34]. Third, we also explored a bifactor model [35] of the TRIRISK model scale. Multidimensional measures often consist of two types of construct-relevant psychometric multidimensionality and these two types are explicitly incorporated in the bifactor model [36].

We used target rotation [34] in the ESEM models. All cross-loadings were specified to be close to zero but not exactly zero, whereas the main factor loadings were freely estimated [36]. The bifactor ESEM was specified with a global risk perception factor alongside three specific factors representing the three different types of risk perception (cf. [36]). The general factor explains variance shared across all items, whereas the specific factors explain item variance unaccounted for by the global factor. To ensure interpretability and adhering to bifactor assumptions, the specific and global factors were specified as orthogonal [35].

We examined longitudinal measurement invariance of the scale to ensure that the same construct was assessed over time. First, a configural model was estimated without constraints. Second, a metric invariance model was specified in which the factor loadings were constrained to be equal over time. Third, a scalar invariance model was estimated where the factor loadings and intercepts were estimated to be equal over time. Fourth, a strict invariance model was estimated where the residual variances, item intercepts, and factor loadings were constrained to equality over time. Decreases in model fit after placing equality constraints on the factor loadings, intercepts, or residual variances, respectively, would indicate measurement non-invariance. We examined the factor structure of the personality dimensions (neuroticism and conscientiousness) in separate ICM-CFA models and included a method factor to account for the potential method effect of having reversed coded items in each scale [37].

To examine the longitudinal relations between risk perception, health behaviors, and personality we estimated two separate path models (*although cross-lagged panel models (CLPM) with autoregressive effects could have been an option with this data and design, recent research indicates that the CLPM is very likely to produce spurious cross-lagged effects when they do not exist, while also underestimating them when they do (Lucas, 2022). Lucas (2022) showed that across varying reliabilities, different ratios of autoregressive to stable-trait variance, and different magnitudes of the autoregressive correlation, severely over- or under-estimated cross-lagged paths are almost guaranteed. Thus, we did not use CLPM due to the high risk of biased estimates*). Risk perception and health behavior variables were measured at two time points (T1 and T2), whereas the personality dimensions were measured only at T1. In the first path model (Model 1), risk perception at T2 was regressed on health behaviors and personality at T1. In the second path model (Model 2), health behaviors at T2 were regressed on risk perception and personality at T1. The control variables (age, gender, relationship status, and prior heart condition or heart surgery) were assessed at T1.

In the correlational analysis and path models we used factor scores [38]. Using factor scores reduces model complexity and the number of freely estimated parameters in the models. Factor scores were deemed more reasonable than including full measurement models given the sample size in the current study. Although factor scores do not provide control for measurement error to the extent full latent variable models do, they provide partial control for measurement error because it gives more weight to items with lower error variance [38]. For the TRIRISK model scale we estimated factor scores based on the most invariant longitudinal model, whereas the factor scores of the personality dimensions were based on the ICM-CFA models.

Model fit was evaluated with conventional fit indices such as the comparative fit index (CFI), the Tucker–Lewis Index (TLI), the standardized root mean squared residual (SRMR), and the root mean square error of approximation (RMSEA). CFI and TLI values around 0.90 and SRMR and RMSEA values around 0.08 indicated acceptable model fit [39]. The longitudinal invariance models were evaluated using Chen’s [40] recommendations that change in CFI (ΔCFI) of less than 0.01 and change in RMSEA (ΔRMSEA) of less than 0.015 or a change in SRMR (ΔSRMR) of less than 0.030 would support metric invariance. For scalar and strict invariance, a change in CFI (ΔCFI) of less than 0.01 and change in RMSEA (ΔRMSEA) of less than 0.015 or a change in SRMR (ΔSRMR) of less than 0.010 would indicate invariance across time. We used MPlus version 8.5 [41] and the full information maximum likelihood estimator to estimate the ICM-CFA, ESEM, bifactor models, and path models. A *p* value of less than 0.05 was used as a criterion that the parameter estimate was statistically significant.

## 3. Results

### 3.1. Measurement Models, Longitudinal Invariance, and Factor Score Estimation

A summary of the model fit indices is shown in Table 1. First, we estimated a three-factor ICM-CFA model of the TRIRISK model scale, which had a poor model fit. Latent factor correlations between the three subscales were 0.568 (affective-deliberate), 0.843 (deliberate-experiential), and 0.908 (affective-experiential) indicating poor discriminant validity among the subscales. Second, we estimated an ESEM of the TRIRISK model scale and compared the model fit and parameter estimates with the ICM-CFA model. The ESEM model provided an acceptable fit to the data and the latent factor correlations were weaker than in the ICM-CFA model, ranging from 0.369 to 0.508. However, items on the experiential subscale had substantial cross-loadings on the affective and deliberate subscales, and the affective subscale had two out-of-bound factor loadings (i.e., larger than 1.0), which suggests that other models should be considered. Third, we estimated a bifactor ESEM model of the TRIRISK model scale that included a global risk perception factor that accounted for shared variance among all of the items and specific factors that explain variance unaccounted for by the global factor.

The ESEM model had an excellent fit to the data and the factor loading pattern showed a well-defined global factor, a relatively well-defined deliberate factor, moderately well-defined affective factor, and poorly defined experiential factor. Furthermore, calculations of omega hierarchical showed that 69% of the reliable variance is due to the global factor, which suggests that most of the test score variance was accounted for by a general risk perception factor [42]. The longitudinal measurement invariance testing of the bifactor ESEM did not show a decreased model fit with increasing constraints (Table 1), suggesting that the same construct was assessed over time.

The ICM-CFA with method factors of neuroticism and conscientiousness showed an adequate model fit (Table 1) and factor scores were estimated based on each measurement model separately. The TRIRISK model scale largely seems to assess a global risk perception factor, and we used factor scores based on the global risk perception factor from most invariant longitudinal bifactor ESEM in the correlational analyses and path models. The factor score determinacy coefficients were 0.930 for neuroticism, 0.901 for conscientiousness, and 0.967 and 0.969 at T1 and T2, respectively, for the global risk perception factor, indicating a strong association between the estimated factor scores and the true factor scores [38,43].

### 3.2. Bivariate Correlations

Correlations between the study variables are displayed in Table 2. Neuroticism was related to higher risk perception and lower fruit/vegetable consumption and less PA. Conscientiousness was related to lower risk perception and higher fruit/vegetable consumption and more PA. Higher risk perception was related to lower fruit/vegetable consumption and less PA.

### 3.3. Path Models

Path models were estimated separately with risk perception as the outcome (Model 1) and health behaviors as outcomes (Model 2). As seen in Model 1 (Table 3), PA did not have a statistically significant effect on risk perception over time (*b* = −0.00, *p* = 0.227); however, both fruit/vegetable consumption (*b* = −0.19, *p* = 0.006) and neuroticism (*b* = 0.22, *p* = 0.001) were statistically significant predictors of risk perception.

As seen in Model 2 (Table 4), risk perception was not a statistically significant predictor of fruit/vegetable consumption (*b* = −0.08, *p* = 0.144) or PA (*b* = −343.86, *p* = 0.147). Neuroticism was a statistically significant and negative predictor of fruit/vegetable consumption (*b* = −0.20, *p* = 0.001) and PA (*b* = −520.84, *p* = 0.029). Higher neuroticism scores were related to less fruit/vegetable consumption and less PA.

## 4. Discussion

The present study represents the first to examine path models involving risk perceptions, health behaviors (PA and fruit/vegetable consumption), and personality traits (conscientiousness and neuroticism). Our first hypothesis was partially supported. Specifically, while risk perceptions did not predict health behaviors, fruit and vegetable consumption predicted risk perceptions in the expected direction. The finding that risk perceptions failed to predict health behaviors contrasts with predictions by several health psychology models, most notably the Health Belief Model which features perceived risk as a key predictor of health behavior [10]. Further, our findings are not concordant with the results of a meta-analysis of experimental studies showing risk perceptions fostered health behavior change [14]. However, it is important to note that only six effect sizes pertained to exercise and five for diet, and the reciprocal associations between the variables were not examined. It is possible that constructs other than risk perceptions may be more powerful predictors of PA and diet and vice versa. Self-regulation [44] or action self-efficacy [45] are examples of other possible candidates. The finding that fruit and vegetable consumption predicted risk perceptions supports the “accuracy hypothesis” that individuals are likely to judge their perceived risk more accurately if they engage in relevant health behaviors [15]. However, this finding did not extend to PA. Therefore, it is possible that participants believed that the consumption of a healthy diet may be more protective of heart health than engagement in PA, although prior research has shown that the behaviors are perceived to be equally important to health [46]. An alternative explanation relates to the measurement of the behaviors. Specifically, while consumption of fruits and vegetables is simple to measure, PA is complex, and it is well known that individuals tend to overestimate their PA [47].

Hypothesis 2 was not supported. Conscientiousness did not predict PA, fruit/vegetable consumption, nor risk perception at T2. Regarding the association with PA and fruit/vegetable consumption, conscientiousness demonstrated small correlations with both health behaviors, but the associations were not present in the final model. Absence of significant effects of conscientiousness on PA and fruit/vegetable consumption were unexpected given the multitude of research labelling conscientiousness as a ‘cardioprotective factor’ due to positive associations with protective behaviors [18,20,21]. This result is likely due to the high stability of the risk perceptions measure in our study [18]. It is also possible that personality plays an increasingly important role over longer periods of time and has accumulating effects on health behaviors. For example, conscientious people may be better at finding ways to initiate *and* maintain health behaviors over time.

Hypothesis 3 was fully supported. Specifically, as expected, neuroticism predicted low levels of PA and fruit/vegetable consumption. Our findings align with previous research suggesting that traits of avoidance, and low impulse control, discipline, and goal directed behavior explain lower engagement in protective health behaviors [18,23]. Individuals high in neuroticism could be counselled through mindfulness to improve impulse control and goal-directed behavior by adopting small health behavior changes, such as a daily walk [48]. Alternatively, the unified protocol, a transdiagnostic treatment regimen, has been shown to significantly reduce neuroticism [49], which could foster greater engagement in health behaviors.

Individuals with high levels of neuroticism perceived themselves to be at higher risk of CVD compared to those with lower levels of this personality trait. This finding aligns with previous suggestions that individuals high in neuroticism perceive themselves at greater risk of disease regardless of their behavior [18]. A direct effect of neuroticism on perceived susceptibility to CVD specifically was also observed in a previous study [19]. We extend the results of Gerent and colleagues who used a four-item comparative measure of perceived risk, by adopting the more comprehensive 18-item TRIRISK measure.

### Strengths and Limitations

Some strengths and limitations of our study should be considered to inform future research. We employed path models, thus allowing temporal sequencing of the variables in our models and assessment of longitudinal measurement invariance of the TRIRISK measure. Further, to address limitations of previous studies which have employed single item measures of risk perceptions, we employed the 18-item TRIRISK measure as it is a more comprehensive assessment of risk perceptions consisting of deliberative, experiential, and affective domains. Although the three-factor model had a poor model fit with poor discriminant validity among the three domains, the use of a multi-item measure of risk perception is a methodological strength of our study. However, unfortunately, we were unable to assess if any specific domain (deliberative, experiential, or affective) was more or less strongly related to the other variables in the model, or if any domain was more or less stable over time.

Limitations of our study also need careful consideration. First, variables other than risk perceptions and personality traits could predict PA and fruit/vegetable consumption. Although risk perception is one of the central variables in the HBM [10], other variables, such as perceived severity, barriers, and benefits were not considered. Further, meta-analytic evidence has shown that risk perception is moderated by response efficacy and self-efficacy, with larger effects on behavior when these are high and response costs are low [14]. Thus, it would be useful in future research to incorporate these and other potential moderators. The one-week time interval between our measurement points may have been too short to observe some of the hypothesized prospective relations (e.g., between risk perceptions and PA), suggesting that longer time intervals should be adopted in future studies. Finally, although we controlled for the existence of heart conditions and prior heart surgery, we did not measure family history of CVD. It is important in future research to examine our research questions in relevant sub-groups, including those with a family history of CVD. It is also recommended that future researchers employ measures that more comprehensively assess all aspects of diet.

## 5. Conclusions

Individuals who consume high levels of fruit/vegetables appear to accurately predict their risk of CVD to be lower than those who consume less fruits/vegetables. No such predictions were evident for PA. Increasing perceptions of risk to CVD do not appear to foster increased engagement in cardio-protective health behaviors (PA and fruit/vegetable consumption). Our results also suggest that high levels of neuroticism in middle age may hinder engagement in PA and consumption of fruit/vegetable behaviors and should therefore be targeted accordingly to increase protective health behaviors and reduce incidence of CVD. Possible future interventions targeting people with high levels of neuroticism could include mindfulness and the unified protocol [49], which could foster greater engagement in health behaviors.

## Figures and Tables

**Table 1:** Model fit indices of the measurement models.

	χ^2^	df	*p*	CFI	TLI	RMSEA	90% CI	SRMR
**TRIRISK model scale**								
ICM-CFA T1	914.37	123	<0.001	0.81	0.78	0.13	[0.13, 0.14]	0.08
ESEM T1	315.84	102	<0.001	0.95	0.92	0.08	[0.07, 0.09]	0.03
Bifactor ESEM T1	177.36	87	<0.001	0.98	0.96	0.06	[0.04, 0.07]	0.02
***Longitudinal invariance***								
Configural	779.49	464	<0.001	0.97	0.96	0.05	[0.04, 0.05]	0.03
Metric	838.64	520	<0.001	0.97	0.96	0.04	[0.04, 0.05]	0.03
Scalar	851.93	534	<0.001	0.97	0.96	0.04	[0.04, 0.05]	0.03
Strict	884.74	552	<0.001	0.97	0.96	0.04	[0.04, 0.05]	0.04
**Personality**								
Neuroticism	92.88	30	<0.001	0.94	0.91	0.08	[0.06, 0.10]	0.04
Conscientiousness	70.81	30	<0.001	0.94	0.91	0.06	[0.04, 0.08]	0.04

**Table 2:** Bivariate correlations among the study variables.

	1	2	3	4	5	6	7	8	9	10	11	12
1. Age												
2. Gender	−0.21 *											
3. Relationship status	−0.01	−0.15										
4. Prior surgery/heart condition	0.10	−0.01	0.14									
5. Neuroticism ^a^	−0.19 *	0.15	−0.13	0.18								
6. Conscientiousness ^a^	0.08	−0.05	0.13	−0.04	−0.49 *							
7. Risk perception T1 ^a^	−0.11	0.03	−0.06	0.38 *	0.34 *	−0.26 *						
8. Risk perception T2 ^a^	−0.10	0.01	−0.06	0.37 *	0.33 *	−0.24 *	0.97 *					
9. Fruit/vegetables T1	0.11	−0.10	0.04	0.10	−0.19 *	0.13 *	−0.19 *	−0.20 *				
10. Fruit/vegetables T2	0.09	0.10	−0.09	0.20	−0.29 *	0.22 *	−0.17 *	−0.18 *	0.48 *			
11. PA T1	0.10 *	−0.08	−0.08	−0.07	−0.15 *	0.19 *	−0.17 *	−0.15 *	0.10 *	0.10		
12. PA T2	0.13 *	−0.14	−0.16	0.00	−0.23 *	0.18 *	−0.17 *	−0.15 *	−0.06	0.08	0.76 *	
*M*	50.61	Na	Na	Na	−0.03	0.02	−0.01	0.00	2.96	2.84	3022	2819
*SD*	6.97	Na	Na	Na	0.94	0.90	0.98	0.97	0.74	0.77	3216	2986
Min	40	Na	Na	Na	1	1	−1.94	−1.91	1	1	0	0
Max	64	Na	Na	Na	5	4.9	2.59	2.50	4	4	19,278	19,278

Note. ^a^ Factor scores, PA = physical activity; T1 = Time 1; T2 = Time 2; * *p* < 0.05.

**Table 3:** Path model with risk perception at T2 as the outcome variable.

	*b* ^a^	*SE*	*p*	*β* ^b^
***Prospective relations***				
Fruit/Vegetables T1 → Risk perception T2	−0.19	0.07	0.006	−0.15
PA T1 → Risk perception T2	−0.00	0.00	0.227	−0.08
Neuroticism → Risk perception T2	0.22	0.07	0.001	0.22
Conscientiousness → Risk perception T2	−0.10	0.07	0.137	−0.10
***Control variables***				
Age → Risk perception T2	−0.01	0.01	0.429	−0.04
Gender → Risk perception T2	−0.09	0.11	0.404	−0.04
Relationship status → Risk perception T2	−0.06	0.12	0.627	−0.03
Prior heart surgery/condition → Risk perception T2	0.77	0.24	0.001	0.17

Note. ^a^ unstandardized estimate, ^b^ standardized estimate, PA = physical activity, gender (0 = male, 1 = female), relationship status (0 = no relation, 1 = in a relation), prior heart surgery/condition (0 = no, 1 = yes).

**Table 4:** Path model with health behaviors at T2 as outcome variables.

	*b* ^a^	*SE*	*p*	*β* ^b^
***Prospective relations***				
Risk perception T1 → Fruit/Vegetables T2	−0.08	0.05	0.144	−0.10
Neuroticism T1 → Fruit/Vegetables T2	−0.20	0.06	0.001	−0.25
Conscientiousness T1 → Fruit/Vegetables T2	0.08	0.06	0.223	0.09
Risk perception T1 → PA T2	−343.86	237.33	0.147	−0.11
Neuroticism T1 → PA T2	−520.84	239.27	0.029	−0.16
Conscientiousness T1 → PA T2	254.28	198.74	0.201	−0.08
***Control variables***				
Age → Fruit/Vegetables T2	0.00	0.01	0.710	0.02
Gender → Fruit/Vegetables T2	0.17	0.11	0.098	0.10
Relationship status → Fruit/Vegetables T2	−0.21	0.12	0.081	−0.11
Prior heart surgery/condition → Fruit/Vegetables T2	0.50	0.20	0.014	0.14
Age → PA T2	25.14	37.88	0.507	0.06
Gender → PA T2	−549.31	462.62	0.235	−0.08
Relationship status → PA T2	−1208.58	567.13	0.033	−0.17
Prior heart surgery/condition → PAT2	602.71	676.65	0.373	0.04

Note. ^a^ unstandardized estimate, ^b^ standardized estimate, PA = physical activity, gender (0 = male, 1 = female), relationship status (0 = no relation, 1 = in a relation), prior heart surgery/condition (0 = no, 1 = yes); T1 = Time 1; T2 = Time 2.

## Data Availability

The data presented in this study are available on reasonable request from the corresponding author. The data are not publicly available as this was not covered by the informed consent of participants.
